# Oral ferric maltol improves iron deficiency anaemia in patients with chronic heart failure

**DOI:** 10.1002/ejhf.3789

**Published:** 2025-07-21

**Authors:** Tibor Kempf, Welf Geller, Jan Fuge, Mircea‐Andrei Sandu, Marius M. Hoeper, Roman Pfister, Sascha Macherey‐Meyer, Stefan Störk, Daniel Scheiber, Jürgen Bogoviku, Jörn Tongers, Tarek Bekfani, Christoph Schindler, Dominik Berliner, Udo Bavendiek, Johann Bauersachs

**Affiliations:** ^1^ Department of Cardiology and Angiology Hannover Medical School Hannover Germany; ^2^ Department of Cardiology and Intensive Care Medicine Städtisches Klinikum Braunschweig Braunschweig Germany; ^3^ Department of Respiratory Medicine and Infectious Diseases Hannover Medical School Hannover Germany; ^4^ Member of the German Center for Lung Research (DZL) Biomedical Research in Endstage and Obstructive Lung Disease Hanover (BREATH) Hannover Germany; ^5^ Faculty of Medicine and University Hospital Cologne, Department III of Internal Medicine University of Cologne Cologne Germany; ^6^ Department Clinical Research & Epidemiology, Comprehensive Heart Failure Center Würzburg University Hospital Würzburg Würzburg Germany; ^7^ Division of Cardiology, Pulmonology and Vascular Medicine, Medical Faculty Heinrich‐Heine University Düsseldorf Germany; ^8^ Division of Cardiology, Angiology and Intensive Medical Care, Department of Internal Medicine I University Hospital Jena Jena Germany; ^9^ Department of Internal Medicine III University Hospital Halle (Saale) Halle Germany; ^10^ Division of Cardiology, Angiology and Intensive Medical Care, Department of Internal Medicine I University Hospital Magdeburg Magdeburg Germany; ^11^ Center for Clinical Trials (ZKS) ‐ Early Clinical Trial Unit (ECTU), Hannover Medical School Hannover Germany

As a frequent comorbidity in patients with heart failure (HF), iron deficiency worsens both symptom severity and overall prognosis.^1^ In patients with HF with reduced ejection fraction (<50%), current guidelines recommend intravenous iron supplementation with ferric carboxymaltose or ferric derisomaltose to improve quality of life, alleviate HF symptoms, and prevent HF hospitalizations.^2^ This recommendation is based on positive trial results with these parenteral iron formulations,^3^ whereas oral iron products were shown to be ineffective and had more side effects. In the IRONOUT‐HF trial, 16 weeks of high‐dose oral iron polysaccharide did not improve exercise capacity in patients with HF with reduced ejection fraction.^4^ Moreover, oral supplementation only minimally increased iron indices, including ferritin and transferrin saturation. Novel oral iron preparations, such as ferric maltol or sucrosomial iron, may have enhanced bioavailability, but clinical trial data are scarce.^5^ Yet ferric maltol is approved for treating iron deficiency and has been shown to be safe, effective, and well tolerated in patients with inflammatory bowel disease, chronic kidney disease, and pulmonary hypertension.^6^, ^7^, ^8^ We therefore conducted a multicentre, open‐label, prospective clinical study, named the ORION‐HF study, to investigate the impact of orally formulated ferric maltol in patients with symptomatic HF and iron deficiency anaemia (EudraCT Number: 2021‐000130‐33). This study evaluated the efficacy of ferric maltol in improving haemoglobin concentration, iron indices, functional capacity, and quality of life in patients with HF and iron deficiency anaemia, in seven German study sites.

ORION‐HF enrolled patients with HF and iron deficiency anaemia, which was defined as either serum ferritin concentration <100 μg/L or 100–299 μg/L with transferrin saturation concentration <20%. Patients were also required to have mild‐to‐moderate anaemia, defined as haemoglobin concentrations ≥8 and <12 g/dl in women and ≥9 and <13 g/dl in men. Eligibility criteria included New York Heart Association (NYHA) class II–IV HF irrespective of left ventricular ejection fraction. The main exclusion criteria were other active haematological disorders, malignancy, active bleeding, ongoing oral or intravenous iron supplementation, concomitant erythropoietin treatment, or severe kidney disease (glomerular filtration rate <20 ml/min). Moreover, patients with either an acute coronary syndrome or a transient ischaemic attack or stroke within the last 30 days were excluded, as were those scheduled to undergo major cardiac or non‐cardiac surgery, device implantation, or percutaneous coronary intervention within the subsequent 16 weeks. The study was approved by the local ethics committee (Nr. 10551_AMG_M_2022), and all patients provided written informed consent. The study was supported by a research grant from Norgine to Hannover Medical School.

Ferric maltol was administered orally at the approved dose of 30 mg twice daily. The primary study objective was the change in haemoglobin levels from baseline to 16 weeks of treatment. Secondary objectives included the effects of oral ferric maltol on iron status (ferritin, transferrin saturation), exercise capacity (6‐min walk test), quality of life (Kansas City Cardiomyopathy Questionnaire [KCCQ]), and safety outcomes.

The study enrolled 50 patients, of whom 41 received at least one dose of the study drug and completed the assessment at 16 weeks (primary analysis as defined per our protocol). The reasons for the nine premature discontinuations were consent withdrawal (*n* = 4) or drop out after an adverse event (*n* = 5). Continuous variables were compared using a paired *t*‐test to assess within‐group differences. To account for multiple comparisons, a Bonferroni correction was applied to 28 tests, setting statistical significance at *p* < 0.00179.

The median age of the patients was 80 (25th–75th percentile, 68–84) years; 32% were women. All patients exhibited symptomatic HF (NYHA class II/III/IV, *n* = 24/4/13) due to HF with either reduced ejection fraction (<50%, *n* = 16) or preserved ejection fraction (≥50%, *n* = 25). Patients had a median left ventricular ejection fraction of 53% (38–62%) and an estimated glomerular filtration rate of 51 (35–63) ml/min/1.73 m^2^. The main results are shown in *Table* *1*. In the primary analysis, oral ferric maltol treatment resulted in significantly increased haemoglobin (from 11.4 [10.9–11.9] to 12.8 [11.8–13.8] g/dl), transferrin saturation (from 14% [8–19] to 29% [22–37]), and ferritin (from 43 [25–82] to 107 [65–140] μg/L); *p* < 0.001 for all comparisons. At baseline, 33 patients (80%) had a transferrin saturation of <20%; among these patients, haemoglobin concentration rose from 11.4 (10.9–11.8) to 13.0 (12.2–14) g/dl at week 16 (*p* < 0.001). In addition, distance walked in the 6‐min walk test improved (from 298 [220–405] to 335 [255–430] m, *p* < 0.001), as did KCCQ overall summary score (from 65 [44–82] to 76 [55–86] score points, *p* = 0.004). Notably, results from the per‐protocol analysis of all patients who completed the 16‐week treatment period (*n* = 35) were entirely consistent (data not shown). As illustrated in *Figure* *1*, pronounced gains in haemoglobin, transferrin saturation, and ferritin occurred within the first 8 weeks of treatment. Online supplementary *Figure* *S1* depicts changes in haemoglobin, iron, transferrin saturation and ferritin from baseline to week 8 or week 16, respectively. Online supplementary *Figure* *S2* illustrates how the severity of iron deficiency relates to the severity of anaemia and the haemoglobin response to iron.

**Table 1:** Results of full analysis

	Baseline (BL)	Week 8	Week 16	*p*‐value BL vs. Week 16
Primary endpoint				
Haemoglobin, g/dl	11.4 (10.9–11.9)	12.7 (11.8–13.4)	12.8 (11.8–13.8)	<0.001
Secondary endpoints				
Iron, μmol/L	9 (5.7–12)	16 (11.2–22)	17.8 (12.4–19.5)	<0.001
Transferrin saturation %	14 (8–19)	25 (20–37)	29 (22–37)	<0.001
Ferritin, μg/L	43 (25–82)	89 (58–130)	107 (65–140)	<0.001
NT‐proBNP, pg/ml	1103 (559–2243)	1140 (588–2385)	1062 (514–2983)	0.675

Data are presented as median and interquartile range. NT‐proBNP, N‐terminal pro‐B‐type natriuretic peptide. Data from 41 patients analysed.

**Figure 1:**
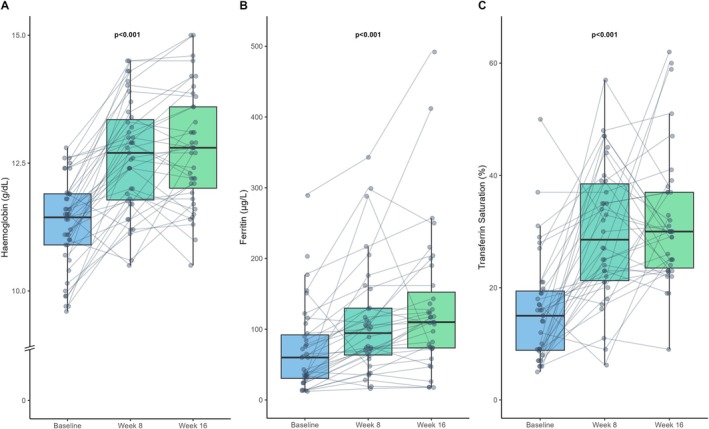
Box plots illustrating the temporal changes in haemoglobin levels (*A*), ferritin concentrations (*B*), and transferrin saturation (*C*) at baseline, 8 weeks, and 16 weeks in all patients from the primary analysis set (*n* = 41). Measurements from baseline to week 16 were compared using a paired *t*‐test.

The ferric maltol treatment was generally well tolerated. Adverse events potentially related to the medication were common (*n* = 19) and predominantly gastrointestinal in nature (e.g. diarrhoea, constipation, flatulence, nausea, and vomiting). Serious adverse events (one case each of pleural empyema, coronary artery disease, retinal vein occlusion, and syncope) were all considered unrelated to the study medication. Two deaths occurred during the main study period, both unrelated to the study medication.

By design, our study has several limitations, including a small sample size, an open‐label design, and a lack of a control group. Additionally, we applied the current European Society of Cardiology HF guideline‐based definition of iron deficiency, despite growing recognition of its limitations. Although dynamic variations in haemoglobin and iron indices have been reported in patients with HF and iron deficiency anaemia,^9^ the consistent and directional changes observed in ORION‐HF indicate that ferric maltol exerts a true pharmacological effect. This result contrasts with those from previous trials with other oral iron formulations, such as oral iron polysaccharide, that did not achieve similar improvements in iron availability.^4^ These results may inform future trials comparing ferric maltol to intravenous iron supplementation in HF.

In conclusion, this observational study in patients with HF found that 16 weeks of ferric maltol was associated with increases in haemoglobin, ferritin, and transferrin saturation, changes that indicate resolution of iron deficiency. Gastrointestinal side effects were common but tolerated by most patients. Whether the observed improvements in symptoms, quality of life, and exercise capacity should be attributed to ferric maltol awaits confirmation in randomized trials comparing it to both placebo and intravenous iron in patients with robustly defined iron deficiency. If ferric maltol and intravenous iron prove to be similarly effective and there is no urgent need to correct iron deficiency, then oral iron supplements would be a logistically less challenging approach to iron supplementation. However, larger, placebo‐controlled trials with longer follow‐up periods are needed to confirm these results and determine the long‐term efficacy and safety of ferric maltol in HF.

### Funding

This work was supported by the German Research Foundation (DFG; Clinical Research Unit [KFO] 311 to J.B., M.M.H., and T.K.). This investigator‐sponsored trial was financially supported by Norgine BV, Amsterdam. The funder had no role in protocol design, study procedures, data acquisition, analysis, or decision to submit for publication.

**Conflict of interest**: T.K. has received honoraria for consultations or lectures from Abbott, AstraZeneca, Vifor, Boehringer Ingelheim, Bayer, Bristol Myers Squibb, Novartis, Roche Diagnostics, Medtronic, Edwards, Norgine, Pharmacosmos, Vifor Pharma, and Sciarc, and has received research support from Vifor Pharma. J.F. has received honoraria for consultations from AstraZeneca. M.M.H. has received honoraria for consultations or lectures from 35Pharma, Acceleron, Actelion, Aerovate, AOP Health, Bayer, Ferrer, Gossamer, Inhibikase, Janssen, Keros, MSD, and Novartis. S.S. has received honoraria for consultations or lectures from AstraZeneca, Bayer, Boehringer Ingelheim, MSD, and Novo Nordisk, all outside the submitted work. T.B. has received honoraria for consultations or lectures from AstraZeneca, Bayer, Boehringer, Pfizer, Pharmacosmos, Sanofi, and Zoll. C.S. has received financial research support, for conducting an investigator‐initiated trial, from Vifor Pharma Intl. AG. U.B. has received travel support and honoraria for lectures/consulting from Alnylam Pharmaceutical, Amgen, Astr Zeneca, Bayer Vital, Bristol Myers Squibb, Novartis, Novo Nordisk, and Pfizer, and institutional research support from Alnylam Pharmaceuticals, all unrelated to this article. J.B. has received honoraria for consultations or lectures from Novartis, Abbott, Bayer, Pfizer, Boehringer Ingelheim, AstraZeneca, Cardior, CVRx, BMS, Amgen, Edwards, Roche, and Zoll, and research support for the department from Zoll, CVRx, Abiomed, Norgine, and Roche. All other authors have nothing to disclose.

## Supporting information


**Appendix S1.** Supporting Information.

## References

[1] Savarese G , von Haehling S , Butler J , Cleland JGF , Ponikowski P , Anker SD . Iron deficiency and cardiovascular disease. Eur Heart J 2023;44:14–27. 10.1093/eurheartj/ehac569 36282723 PMC9805408

[2] McDonagh TA , Metra M , Adamo M , Gardner RS , Baumbach A , Böhm M, et al. 2023 Focused Update of the 2021 ESC Guidelines for the diagnosis and treatment of acute and chronic heart failure: Developed by the task force for the diagnosis and treatment of acute and chronic heart failure of the European Society of Cardiology (ESC). With the special contribution of the Heart Failure Association (HFA) of the ESC. Eur J Heart Fail 2024;26(1):5–17. 10.1002/ejhf.3024 38169072

[3] Anker SD , Karakas M , Mentz RJ , Ponikowski P , Butler J , Khan MS , *et al*. Systematic review and meta‐analysis of intravenous iron therapy for patients with heart failure and iron deficiency. Nat Med 2025;31:2640–2646. 10.1038/s41591-025-03671-1 40159279 PMC12353798

[4] Lewis GD , Malhotra R , Hernandez AF , McNulty SE , Smith A , Felker GM , *et al*.; NHLBI Heart Failure Clinical Research Network . Effect of oral iron repletion on exercise capacity in patients with heart failure with reduced ejection fraction and iron deficiency: The IRONOUT HF randomized clinical trial. JAMA 2017;317:1958–1966. 10.1001/jama.2017.5427 28510680 PMC5703044

[5] Karavidas A , Troganis E , Lazaros G , Balta D , Karavidas IN , Polyzogopoulou E , *et al*. Oral sucrosomial iron improves exercise capacity and quality of life in heart failure with reduced ejection fraction and iron deficiency: A non‐randomized, open‐label, proof‐of‐concept study. Eur J Heart Fail 2021;23:593–597. 10.1002/ejhf.2092 33421230

[6] Gasche C , Ahmad T , Tulassay Z , Baumgart DC , Bokemeyer B , Büning C , *et al*.; AEGIS Study Group . Ferric maltol is effective in correcting iron deficiency anemia in patients with inflammatory bowel disease: Results from a phase‐3 clinical trial program. Inflamm Bowel Dis 2015;21:579–588. 10.1097/MIB.0000000000000314 25545376 PMC4342319

[7] Olsson KM , Fuge J , Brod T , Kamp JC , Schmitto J , Kempf T , *et al*. Oral iron supplementation with ferric maltol in patients with pulmonary hypertension. Eur Respir J 2020;56:2000616. 10.1183/13993003.00616-2020 32444411 PMC7676873

[8] Pergola PE , Kopyt NP . Oral ferric maltol for the treatment of iron‐deficiency anemia in patients with CKD: A randomized trial and open‐label extension. Am J Kidney Dis 2021;78:846–856. 10.1053/j.ajkd.2021.03.020 34029682

[9] Graham FJ , Masini G , Pellicori P , Cleland JGF , Greenlaw N , Friday J , *et al*. Natural history and prognostic significance of iron deficiency and anaemia in ambulatory patients with chronic heart failure. Eur J Heart Fail 2022;24:807–817. 10.1002/ejhf.2251 34050582

