# Supplementary figures and images for: Oral ferric maltol improves iron deficiency anaemia in patients with chronic heart failure
Source: Eur J Heart Fail. 2025 Jul 21;27(11):2325–7. doi: 10.1002/ejhf.3789 (PMC12765365; doi:10.1002/ejhf.3789)

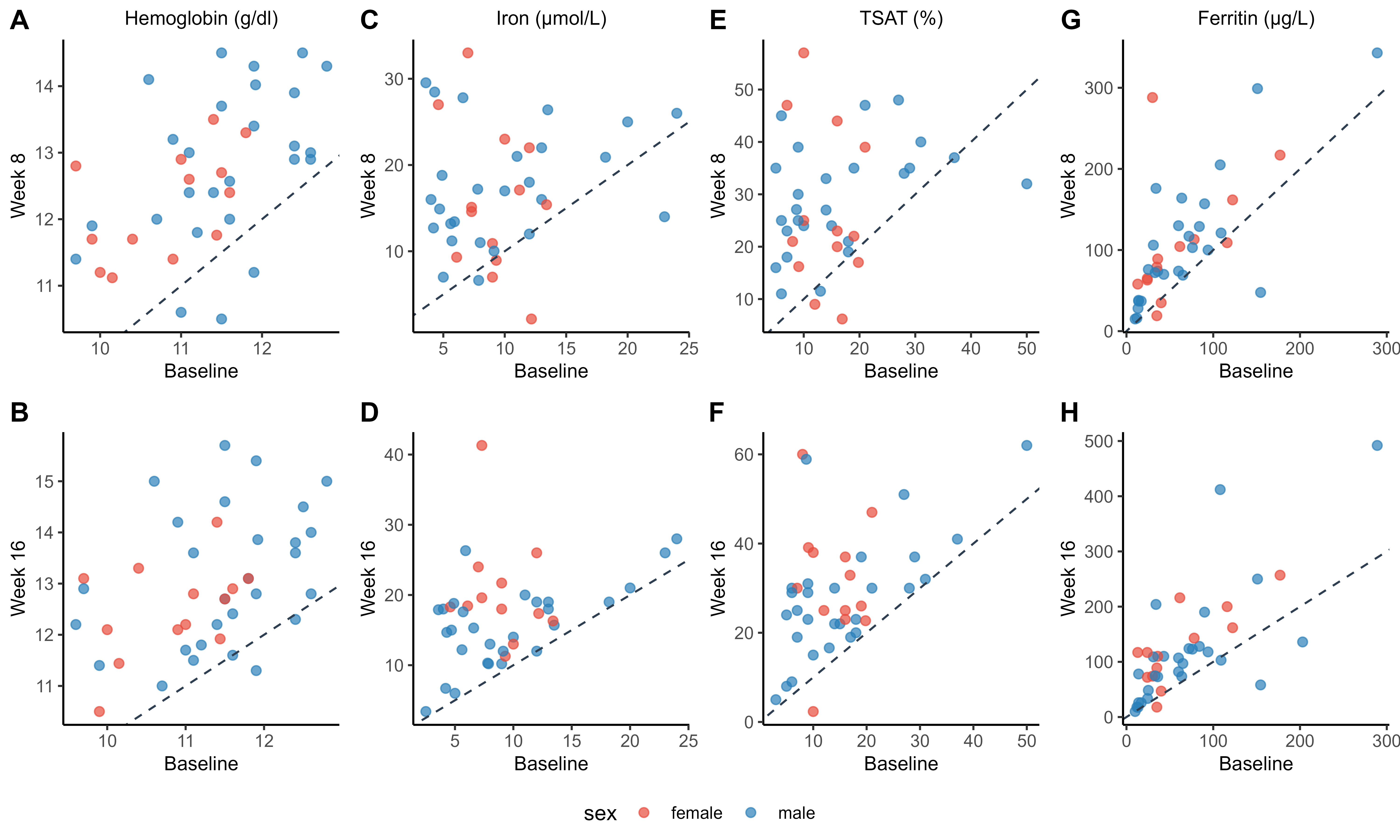

Supplement: Supplementary file 1 — Appendix S1. Supporting Information. [file EJHF-27-2325-s001.zip › ejhf3789-sup-0001-FigureS1.png]

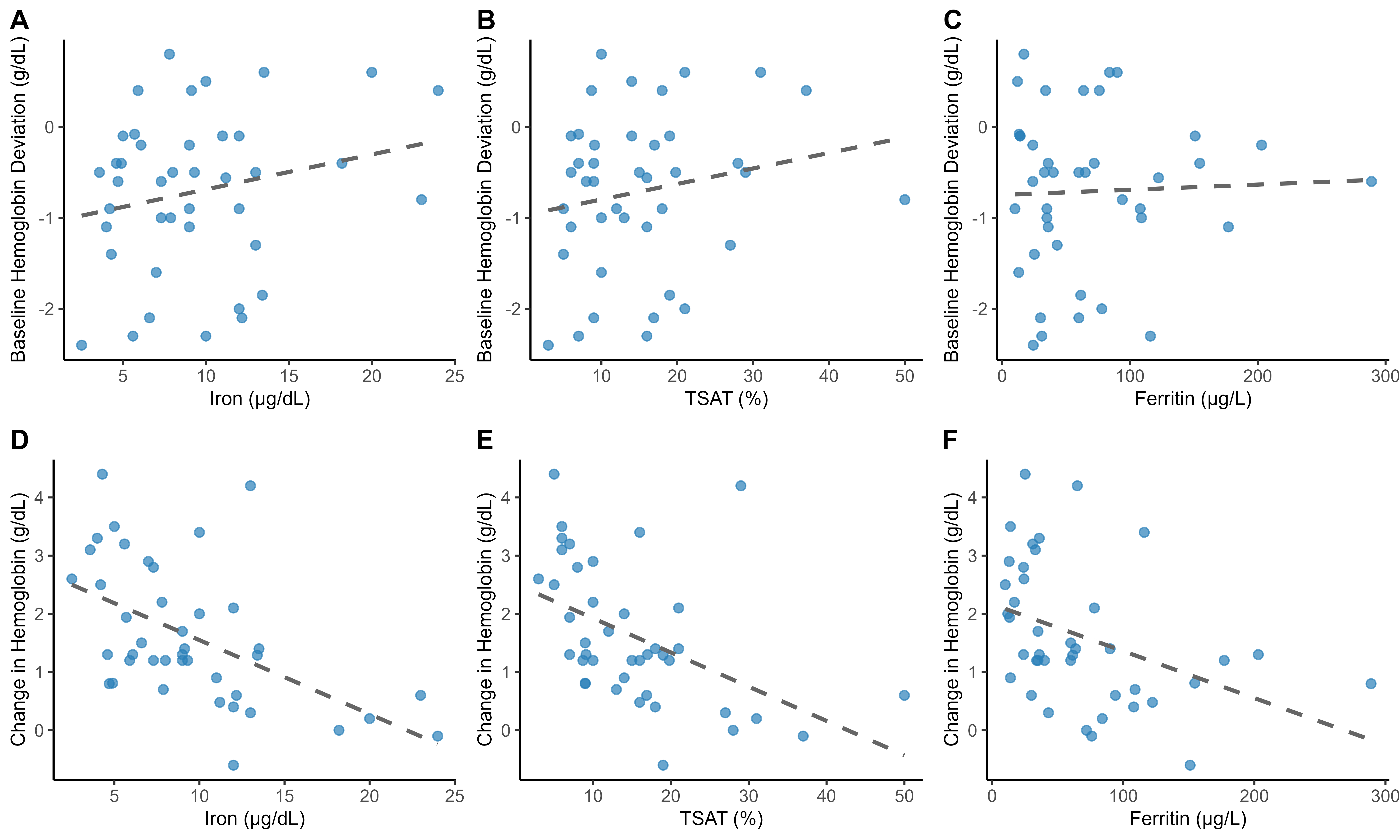

Supplement: Supplementary file 1 — Appendix S1. Supporting Information. [file EJHF-27-2325-s001.zip › ejhf3789-sup-0002-FigureS2.png]
